# Laparoscopic versus open gastrectomy for locally advanced gastric cancer: a systematic review and meta-analysis of randomized controlled studies

**DOI:** 10.1186/s12957-019-1600-1

**Published:** 2019-04-15

**Authors:** Katharina Beyer, Ann-Kathrin Baukloh, Carsten Kamphues, Hendrik Seeliger, Claus-Dieter Heidecke, Martin E. Kreis, Maciej Patrzyk

**Affiliations:** 1 Klink für Allgemein-, Viszeral- und Gefäßchirurgie, Charité – Universitätsmedizin Berlin, Campus Benjamin Franklin, Hindenburgdamm 30, 12200 Berlin, Germany; 2 Klinik für Allgemein-, Viszeral-, Thorax- und Gefäßchirurgie, Universitätsmedizin Greifswald, Sauerbruchstraße, 17475 Greifswald, Germany

## Abstract

**Background:**

This meta-analysis sought to evaluate the potential benefits and harms of laparoscopic gastrectomy with D2 lymphadenectomy for locally advanced gastric cancer versus open surgery.

**Methods:**

A comprehensive search for randomized controlled studies that compared laparoscopic versus open gastrectomy with D2 lymphadenectomy for locally advanced gastric cancer published until December 31, 2018, was conducted. Operative outcomes, early postoperative outcomes, and long-term results were analyzed using a random effects model.

**Results:**

Five randomized controlled trials containing a collective total of 2157 patients were included. In comparison with open surgery, laparoscopic gastrectomy for locally advanced gastric cancer showed similar risks of short-term mortality and serious adverse events within 30 days after surgery. Regarding intraoperative outcomes, operative time was increased for the laparoscopic approach, whereas the estimated intraoperative blood loss tended to be less. However, the amount of evidence was low for most outcomes. In addition, the results for the length of hospital stay and time to first flatus did not show statistically significant differences. The number of harvested lymph nodes and compliance with D2 lymphadenectomy did not significantly differ between the two groups, indicating oncological equivalence of both approaches. However, long-term oncological results could not be evaluated due to a lack of relevant data in four of the trials.

**Conclusion:**

Laparoscopic gastrectomy with D2 lymphadenectomy can be performed with equivalent overall short-term morbidity and mortality versus the open approach for locally advanced gastric cancer. However, further well-designed randomized controlled trials are necessary to assess the possible advantages and risks of the laparoscopic approach as well as the long-term results.

**Electronic supplementary material:**

The online version of this article (10.1186/s12957-019-1600-1) contains supplementary material, which is available to authorized users.

## Introduction

Kitano first performed laparoscopy-assisted distal gastrectomy for early gastric cancer in 1994 [1], and since then, this minimally invasive approach has grown increasingly popular. Almost a quarter of a century later, minimally invasive surgery for gastric cancer is being carried out more and more frequently in Asia and the Western world, though most experiences with minimally invasive surgery for gastric cancer come from Asia due to the higher disease incidence in this region. In Korea and Japan specifically, nationwide surveillance programs have played a role in the increased detection of gastric cancer at early stages [2], resulting in a substantial amount of experience with minimally invasive surgery for early gastric cancer. Several previous randomized controlled trials (RCTs) compared laparoscopic to open surgery for the treatment of early gastric cancer. Several meta-analyses of the data from these RCTs and from high-quality nonrandomized trials suggested the minimally invasive approach results in less blood loss and decreased complication rates [3, 4]. However, both previous meta-analyses also indicated that the number of harvested lymph nodes was reduced and operative time was increased in conjunction with the laparoscopic approach. Separately, a recent Cochrane Review including RCTs compared laparoscopic versus open gastrectomy for gastric cancer and found no evidence for any differences in long- or short-term outcomes between the two approaches [5], although this determination was based on a very low level of evidence.

With increasing surgical experience, indications for laparoscopic surgery have been extended to locally advanced tumor stages. Because the completeness of lymphadenectomy becomes more important with increases in tumor stage, it is important to determine whether or not the minimally invasive approach is equivalent to the open approach for advanced gastric cancer.

The aim of this systematic review and meta-analysis was therefore to identify and analyze RCTs in order to compare the short- and long-term outcomes of open versus laparoscopic gastrectomy with D2 lymphadenectomy in adults suffering from locally advanced gastric cancer.

The Patient/Problem/Population, Intervention, Comparison/Control/Comparator, Outcome (PICOS) criteria for the inclusion and exclusion of studies are provided in “Appendix 1.”

## Materials and methods

The present meta-analysis was performed according to a protocol and the Preferred Reporting Items for Systematic Reviews and Meta-Analyses (PRISMA) guidelines. The protocol of this meta-analysis can be obtained upon request. The PRISMA checklist is provided in Additional file 1.

### Search strategy

The primary aim of this meta-analysis was to compare the outcomes of open versus laparoscopic gastrectomy with D2 lymphadenectomy for locally advanced gastric cancer.

A comprehensive literature search was conducted to identify all trials dealing with laparoscopic gastrectomy for gastric cancer. Therefore, searches in the MEDLINE (PubMed), EMBASE (Ovid), and CENTRAL databases were performed. Additionally, the search results were broadened by browsing the references of the retrieved articles. All publications with full-text versions available published from January 1, 1980, to December 31, 2018, were included in the search.

To obtain all studies dealing with a laparoscopic or laparoscopy-assisted approach for gastric cancer, a search was performed with the key words Gastrectomy AND (gastric OR stomach) AND (cancer OR carcinoma) AND (laparosc* OR minimally-invasive).

The search strategy is stated in “Appendix 2.”

The results were copied into EndNote X9 (Clarivate Analytics, Philadelphia, PA, USA), and duplicates were identified and removed. Additionally, the results were manually screened for duplicates.

Among the remaining studies, trials comparing laparoscopic to open gastrectomy were subsequently identified via the manual screening of titles and abstracts.

Full-text versions of the resulting publications were reviewed to identify RCTs including patients with locally advanced gastric cancer.

### Eligibility criteria

Randomized clinical trials comparing open to laparoscopic and/or laparoscopy-assisted gastrectomy in adults with locally advanced gastric adenocarcinoma were included. Thus, adults undergoing gastrectomy with D2 lymphadenectomy for locally advanced gastric cancer were included. Patients younger than 18 years old were excluded.

Locally advanced gastric cancer was defined as histologically proven, nonmetastatic gastric cancer with a pretherapeutic stage equal to or greater than an American Joint Committee on Cancer/Union for International Cancer Control stage 2. Studies including early and locally advanced gastric cancer cases were not excluded unless they lacked separate outcome reports for patients suffering from advanced gastric cancer.

Patients undergoing gastrectomy for reasons other than gastric adenocarcinoma such as gastrointestinal stroma tumors, neuroendocrine tumors, or benign lesions were excluded. However, trials with these entities were included if they contained separate outcome reports for locally advanced gastric cancer.

With regard to the extent of surgery, both total and subtotal and distal as well as proximal resections were included. However, a D2 lymphadenectomy was required.

Studies reporting results of hand-assisted minimally invasive gastrectomy were excluded. Additionally, trials comparing open to robotic gastrectomy were excluded unless they were three-armed studies that included a laparoscopic arm.

Studies that contained at least one of the following primary outcome measures were included*:* short-term mortality, anastomotic leakage, postoperative serious adverse events within 30 days after surgery, length of hospital stay, and health-related quality of life within three and 12 months after surgery. The definition of serious adverse events was accepted as a Clavien–Dindo grade 3 or greater [6], or as defined by the Accordion Severity Classification of Postoperative Complications (ASCPC) classification system [7].

Secondary outcomes included incision-to-closure time, estimated intraoperative blood loss, intraoperative complications, number of harvested lymph nodes, compliance with D2 lymphadenectomy, any type of postoperative complication, frequency of wound complications, frequency of pulmonary complications, frequency of revision surgery, time to first flatus, return to normal life activity, and return to work. Overall survival at the maximum follow-up point, 3-year survival, recurrence rate, and 3-year disease-free survival were additional secondary outcomes.

Postoperative complications were defined as any adverse event occurring within 30 days following surgery.

Studies with a mean follow-up period of less than 30 days were excluded.

For the systemic review as well as for the meta-analysis, only RCTs were included. PICOS criteria for inclusion and exclusion are provided in “Appendix 1.”

Regarding report characteristics, only English-language publications that were available as full-text versions and published before December 31, 2018, were included. Publications that were published as online ahead of print publications were also included. Trials that were either not published in English or that had not yet been published as full-text versions were excluded.

### Study selection and data extraction

First, the titles and abstracts of all retrieved articles were separately reviewed by two investigators. For all articles that were eligible or potentially eligible or where eligibility was unclear, full-text versions were assessed to establish eligibility. Trials with full-text versions not published in English were excluded. Then, two investigators independently screened the full-text versions and determined final inclusion of the articles by consensus.

**Fig. 1:**
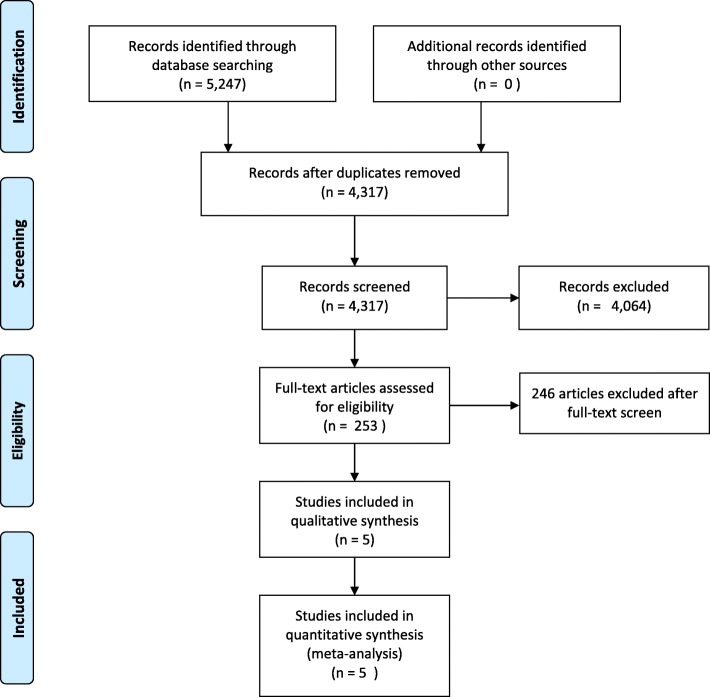
Study flow diagram

The selection process was recorded to complete a PRISMA flow diagram (Fig. 1).

Data were extracted using standardized forms, collecting the study design, total study duration, number of study centers and locations, study settings, withdrawals, and date(s) of study. Furthermore, the number of participants, mean age, age range, gender, American Society of Anesthesiologists (ASA) status, inclusion criteria, and exclusion criteria were recorded for each type of intervention. Primary and secondary outcomes were specified and collected. Additionally, funding and notable conflicts of interest of the trial authors were retrieved.

Data were copied into Excel spreadsheets (Microsoft Corp., Redmond, WA, USA) and were double-checked for mistakes.

### Quality assessment/risk of bias in individual studies

Two investigators independently assessed the risk of bias for each study using the criteria outlined in chapter 8 of the *Cochrane Handbook for Systematic Reviews of Interventions*.

Disagreements were resolved by discussion. The risk of bias was assessed in the aspects of random sequence generation, allocation concealment, blinding of participants and personnel, blinding of outcomes assessment, incomplete outcomes data, selective outcomes reporting, and other biases.

Each aspect of bias assessment was graded as demonstrating a high, low, or unclear risk of bias for each study. The risk of bias judgements was summarized across different studies for each of the respective aspects. Risk of bias was visualized using R and the package “ggplot2” [8].

We considered trials that showed a low risk of bias in all domains to have an overall low risk of bias. Other trials were considered to have an unclear or high risk of bias.

### Statistical analysis

This meta-analysis was performed according to current recommendations from the Cochrane Collaboration and the Quality of Reporting of Meta-Analyses guidelines.

R [8] with the meta and the metafor package [9] as well as Comprehensive Meta-Analysis Software Version 3.0 (Biostat, USA) were used for statistical analyses.

Dichotomous data were analyzed as risk ratios (RRs) with 95% confidential intervals (CIs). For continuous variables, mean differences (MDs) with 95% CIs were used when the outcomes were reported or converted into the same units across all of the included studies. When several units for outcome measurements were given among the trials, standardized mean differences and 95% CIs were used. For analyses employing dichotomous and continuous variables, odds ratios (ORs) and 95% CIs were calculated. Prediction intervals were determined using R [8] with the packages “metafor” and “meta.”

A random-effects model was used to analyze all outcomes, as we assumed that the treatment effect may vary among the studies due to differences in the inclusion and exclusion criteria as well as in surgical quality controls.

We used the tau^2^ and *I*^2^ statistics to measure statistical heterogeneity among the trials for each analysis. To identify sources of significant heterogeneity, sensitivity analyses were performed by removing individual studies from the dataset and analyzing the effect on the overall results. Additionally, Baujat and L’Abbe plots were calculated.

Because less than 10 trials were included in this analysis, the creation of a funnel plot to explore publication bias was not meaningful.

Results were summarized in Forest plots.

### Summary of findings’ table

The quality of evidence was assessed for each outcome using the Grading of Recommendations Assessment, Development, and Evaluation (GRADE) approach. “High-quality” evidence was downgraded in the case of serious (one level) or very serious (two levels) limitations depending on the outcomes of the risk of bias, inconsistency of results, indirectness of evidence, and imprecision or publication bias assessments. Thus, the evidence level could be downgraded from high to moderate (one level) and low (two levels) to very low (three levels). A summary of findings table was created using the GRADEpro GDT (Evidence Prime, Inc.) software.

## Results

### Results of publication search

The search strategy revealed 5247 publications. After removing duplicates, 4317 publications remained. Of these, 253 publications compared minimally invasive gastrectomy for gastric cancer to the open approach and were retrieved to assess further. Of these studies, 232 were excluded, as they did not include patients with advanced gastric cancer, were reports of nonrandomized trials, or were protocols of ongoing studies. Two publications did not provide any data for a control group [10, 11] and were thus also excluded. A third study [12] compared open gastrectomy to hand-assisted laparoscopic surgery and was thus excluded.

Of the remaining 18 publications, six [13–18] included reports of patients with early and advanced gastric cancer. Two of the reports were generated from patients of the same study population [16, 17], and two reports covered the same trial [13, 14].

All trials that included patients with early and advanced gastric cancer did not report data from the advanced cancer patients separately, and these studies were therefore excluded from the present meta-analysis.

The remaining 12 publications included six full-text publications [19–25] and six abstracts of presentations held at annual meetings [26–30]. Of these, two congress abstracts reported data included in respective full-text publications of the same trial [26, 27]. The remaining three abstracts referred to trials that have not yet been published in full and were thus excluded. Of the six full-text publications, one contained the protocol of a study that had already been published in full text [21]. Therefore, five publications of five trials remained. Figure 1 shows the study selection process used in the present meta-analysis.

### Studies included in this meta-analysis

A total of 2157 patients suffering from locally advanced gastric cancer in four two-armed trials comparing the minimally invasive procedure to an open approach were included in this meta-analysis. Table 1 provides an overview of the trials included in this meta-analysis. Additionally, inclusion and exclusion criteria of the trials included in this meta-analysis are given in “Appendix 3.”

**Table 1:** An overview of the trials included in the meta-analysis

Author	References	Country	*N* (postrandomization drop outs *N* (%))	Type of intervention			Outcome
				Comparator /control	LAD	Extend of resection	
Cai	Cai et al. [19]	China Single-center	123 (27 (22%))	LAG/OG	D2	PG DG TG	Operative outcomes: operating time, intraoperative blood transfusion, estimated blood loss, tumor size, number of lymph nodes harvested, frequency of open conversion Postoperative recovery: days with a body temperature > 37 °C, time to first flatus, number of days to get out of bed, time to first liquid intake, hospital stay Postoperative morbidity: morbidity rate, type, and frequency of postoperative complications Postoperative mortality Long-term results: survival at mean follow-up
Hu	Hu et al. [22] Hu et al. [26]	China Multi-center	1056 (17 (2%))	LAG/OG	D2	DG	Operative outcomes: operating time, estimated blood loss, frequency of open conversion, intraoperative blood transfusion, proximal resection margins, distal resection margins, number of lymph nodes harvested, length of incision, intraoperative complications Postoperative recovery: time to ambulation, time to first flatus, time to first liquid intake, postoperative hospital stay Postoperative morbidity: severity and frequency of postoperative complications (Clavien–Dindo), type of postoperative complications Postoperative mortality Long-term results: 3-year DFS (primary outcome, results pending)
Park	Park et al. [20] Kim et al. [23] Kim et al. [21] Nam et al. [23]	Korea Multi-center	204 (8 (4%))	LAG/OG	D2	DG	Operative outcomes: Noncompliance rate to nodal dissection (primary outcome), number of lymph nodes harvested, operation time, resectability, intraoperative complications, Postoperative recovery: time to first liquid intake, length of hospital stay, inflammatory response markers, delivery of adjuvant chemotherapy Postoperative morbidity: severity and frequency of postoperative complications (ASCPC) Postoperative mortality Long-term results: 3-year DFS
Shi	Shi et al. [24]	China Single-center	328 (6 pre-randomization)	LAG/OG	D2	PG DG TG	Operative outcomes: operation time, estimated blood loss, intraoperative blood transfusion, number of lymph nodes harvested, frequency of open conversion Postoperative recovery: analgesic injection use, time to first flatus, time to first liquid intake, time to ambulation, postoperative hospital stay, Postoperative morbidity: severity and frequency of postoperative complications (Clavien–Dindo), type of postoperative complications, frequency of reoperation Postoperative mortality Long-term results: 5-year OS (primary outcome, results pending)
Wang	Wang et al. [25]	China Multi-center	446 (4 (1%))	LAG/OG	D2	DG	Operative outcomes: operating time, estimated blood loss, frequency of open conversion, intraoperative blood transfusion, proximal resection margins, distal resection margins, number of lymph nodes harvested, length of incision, intraoperative complications Postoperative recovery: time to ambulation, time to first flatus, time to first liquid intake, postoperative hospital stay Postoperative morbidity: overall postoperative morbidity (primary outcome), severity and frequency of postoperative complications (Clavien–Dindo), type of postoperative complications Postoperative mortality Long-term results: 3-year DFS (results pending)

*PG* proximal gastrectomy, *DG* distal gastrectomy, *TG* total gastrectomy, *LAG* laparoscopic-assisted gastrectomy, *OG* open gastrectomy, *LAD* lymphadenectomy

Three of the included trials were multicenter studies [22, 23, 25], whereas the remaining trials were performed in a single center.

All trials included patients suffering from histologically proven adenocancer of the stomach. The trials reported by Hu et al., Park et al., and Wang et al. [22, 23, 25] included patients with clinical tumor stages cT2 to 4a, N0 to N3, and cM0, whereas the trial reported by Shi excluded patients with a T-stage higher than T3. Cai et al. [18] included patients suffering from early, locally advanced, or metastatic gastric cancer. Outcomes of patients with locally advanced nonmetastastic gastric cancer were selectively reported in this study.

All trials excluded patients with a history of major upper abdominal surgery as well as patients with an ASA score of more than 3. Patients aged younger than 18 years of age were excluded in the trials reported by Hu et al., Park et al., Shi et al., and Wang et al., whereas Cai et al. did not indicate whether any age groups had been excluded from study participation.

In the trial reported by Shi et al., six patients were excluded prior to randomization because resection had not been performed [24]. This exclusion was the result of the study design indicating that all patients underwent laparoscopic exploration, and randomization occurred only when resectability had been confirmed. Therefore, of the 2157 patients included in this meta-analysis, 2151 patients had been randomized and 1079 (50.2%) were assigned to minimally invasive resection and 1072 (49.8%) to open resection. Of these, 56 patients (2.6%) were excluded after randomization. Of these cases, 27 (2.5%) cases were in the laparoscopic group and 29 (2.7%) cases in the open group.

Patients were excluded from the trials reported by Hu et al., Park et al., and Wang et al. for not meeting the inclusion criteria (*n* = 8), refusing to participate (*n* = 6), or due to irresectability (*n* = 15). The remaining cases were analyzed using a modified intention-to-treat analysis in all three studies.

The trial reported by Cai et al. [19] included patients suffering from early, locally advanced, or metastatic gastric cancer. Following randomization, nine patients with early and nine patients with metastatic gastric cancer were excluded. The two remaining cases had been excluded from the trial reported by Cai et al. due to a necessary conversion to an open approach [19]. In the remaining studies, necessary conversion cases remained within the laparoscopic group and thus were not excluded.

In summary, the data of 1052 patients treated with laparoscopic gastrectomy and the data of 1043 patients treated with the open approach were presented within the included trials.

In the trials reported by Cai et al., Park et al., Shi et al., and Wang et al. [19, 23–25], all minimally invasive surgery was conducted as laparoscopy-assisted surgery; in the remaining trial [22], laparoscopy-assisted surgery was recommended, but totally laparoscopic surgery was also allowed depending on the surgeon’s preference.

D2 lymphadenectomy was the standard procedure in each of the included trials.

Whereas the trials reported by Hu et al. (CLASS-01, [22]), Park et al. (COACT 1001, [23]), and Wang et al. only included patients for whom distal gastrectomy was planned, the trials reported by Cai et al. and Shi et al. also included patients with planned total and proximal gastrectomy [19, 24]. Therefore, most patients were treated by distal gastrectomy. Within the minimally invasive group, 929 (86.1%) received distal gastrectomy, 33 (3.1%) received proximal gastrectomy, and 90 (8.3%) patients were treated by total gastrectomy. The remaining cases (*n* = 27, 2.5%) were excluded following randomization, and for these patients, data were not provided on the type of surgery performed.

Conversion to the open approach was necessary in 57 (5.3%) of the 1079 cases randomized to minimally invasive surgery. Patients who underwent conversion were excluded from further analysis in one trial [19].

Within the open group, 40 (3.7%) patients received proximal gastrectomy, 924 (86.2%) were treated by distal gastrectomy, and 78 (7.3%) patients received total gastrectomy. One patient (0.1%) only underwent a biopsy due to irresectability of the tumor. The remaining 29 patients (2.7%) were excluded following randomization.

In one trial, anastomosis was conducted by stapling [24], whereas the remaining trials did not provide information on anastomosis.

Drains were routinely used in one trial [24], whereas information on drain use was not provided in the remaining trials.

In four of the five studies [22–25], surgical quality was assured by reviewing video records and photographs of the surgical site. In the trial published by Park et al., completeness of D2 lymphadenectomy was evaluated using a list of checkpoints when reviewing video records [23]. Cai et al. did not report a quality control. This may be due to the fact that only three surgeons participated in this study and only one surgeon performed laparoscopic gastrectomy [19].

The amount of laparoscopic gastrectomies performed previously by every single surgeon differed between studies: for example, while the trial reported by Wang included only surgeons who had performed at least 60 laparoscopic and 60 open gastrectomies, the study reported by Park required only 30 laparoscopic distal gastrectomies (“Appendix 4”) [23, 25].

In the trials reported by Hu et al., Park et al., Shi et al., and Wang et al., the included patients did not receive preoperative chemotherapy or radiochemotherapy. However, this information was not provided for the trial reported by Cai et al. Also, in the trials reported by Hu et al. and Shi et al., adjuvant chemotherapy was administered as per the protocol in the absence of contraindications. The rate of patients who received chemotherapy was not provided [22]. Park et al. reported that adjuvant chemotherapy was given in all patients with a tumor stage of more than grade 2. The number of patients who actually received chemotherapy did not show a statistically significant difference between the study groups (*p* = 0.559) [23].

The mean follow-up period was 22 months [19] in the trial reported by Cai et al. and 38 months in the study reported by Park et al [23]. For the remaining trials, the mean follow-up time was not provided. This is due to the fact that the follow-up in these studies has not yet been completed, because the long-term data are still collected.

### Risk of bias in included studies

**Fig. 2:**
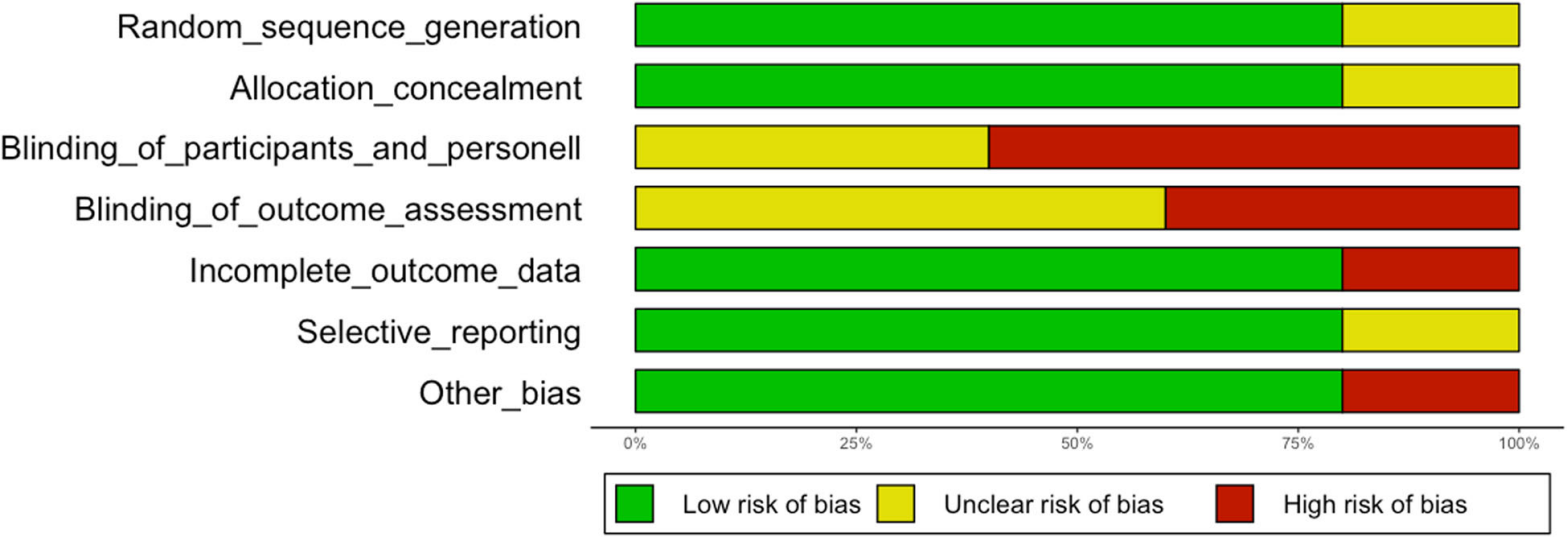
Risk of bias graph presenting review authors’ judgements about each risk of bias item as percentages across all included studies

Selection bias was of an unclear level of risk in the trial reported by Cai et al. as information on random sequence generation, and allocation concealment was not available (Fig. 2). However, baseline characteristics did not indicate an imbalance. Each of the remaining trials showed a low risk of selection bias.

Three of the five trials did not describe blinding measures within the full-text publications. However, within the study protocol of the study reported by Hu et al., which is available at www.clinicaltrials.gov, it was stated that blinding methods had not been used. Thus, the risks of performance and detection bias were both high.

Shi et al. indicated in the study protocol published at www.clinicaltrials.gov that they planned to blind both study participants and study staff in their study. However, the publication of the results lacks a description of the blinding methods, so that the risk of bias remains unclear.

Park et al. stated that blinding methods had not been used within their trial. However, within the study protocol of their study, it was stated that blinded assessment of the primary outcome (i.e., compliance to D2 lymphadenectomy) would be provided by blinded observers, whereas the patients were not blinded [21]. Thus, the risk for performance bias was high and detection bias was of unclear risk.

Wang et al. reported that blinding methods were not used within their trial. Therefore, the risks for performance and detection bias were both high in the study reported by Wang et al.

As there were 22% postrandomization dropouts, the risk of attrition bias was high in the study reported by Cai et al. Particularly critical is the fact that the patients who needed an open conversion were excluded from the analysis. In contrast, the number of postrandomization dropouts was low and a modified intention-to-treat analysis was used in each of the remaining trials. Therefore, there was a low risk of attrition bias within the remaining four studies.

Study protocols were not available for the trials reported by Cai et al. and did thus display an unclear risk of reporting bias. The respective protocols of the trials reported by Hu et al., Park et al., Shi et al., and Wang et al. are published at clinicaltrials.gov. With the exception of the study published by Park et al., all other three publications present only short-term results, while long-term results are still pending. The short-term results were recorded according to the study protocol. Therefore, the risk for reporting bias is low in these publications.

In addition, another source of bias was identified having to do with the qualification of participating surgeons, as the criteria for the selection of the participating surgeons differed between the individual studies. The number of procedures required to be performed by a surgeon to master laparoscopic gastrectomy is a subject of an ongoing debate. According to the literature, it can be assumed that learning curves have been completed after performing anywhere from 40 to 90 laparoscopic resections [23, 30, 31]. However, the number of laparoscopic gastrectomies that must be performed to reach a plateau seems to depend upon the annual case load of the surgeon’s institution [32] as well as on each surgeon’s skills and experience with laparoscopic surgery.

Whereas surgeons had to have performed at least 50 distal gastrectomies with D2 lymphadenectomy using open and laparoscopic approaches in the trials reported by Hu et al. and Shi et al., the trial reported by Park et al. required only an experience of 30 laparoscopic gastrectomies. However, in the trial reported by Hu et al., surgeons were determined to be qualified by the CLASS academic committee on the basis of the evaluation of unedited videos of both their open and laparoscopic gastrectomy with D2 lymphadenectomy procedures. Additionally, only surgeons with at least 300 gastrectomies for patients with advanced gastric cancer annually at each institute participated, indicating a very high case load for every single surgeon. Therefore, the overall risk of bias due to learning curve issues was low in this trial.

Within the study reported by Shi et al., surgical quality was assessed by regular reviews of videos in minimally invasive surgery and photographs in open surgery.

In the trial reported by Park et al., completeness of D2 lymphadenectomy was observed by evaluating uncut videos using a checklist. However, compliance with D2 lymphadenectomy was a primary outcome and not a tool to evaluate the quality of individual surgeons in this study. A minimum annual amount of gastrectomies for participating institutions was not required in this study.

The highest number of previously performed gastrectomies was required in the study reported by Wang et al., as they required at least 60 laparoscopic and 60 open procedures.

In view of these facts, it can be summed up that all of the four studies discussed took measures to minimize the effects of an ongoing learning curve. Above all, these measures concern the control of the quality of D2 lymphadenectomy.

In light of the conflicting learning curve data above, it cannot be ruled out completely that all participating surgeons had completed the learning curve at the time of their respective study inclusion. Nevertheless, this risk primarily affects technically difficult aspects beyond lymphadenectomy such as anastomosis. However, the overall risk of bias is low for this domain in the trials reported by Hu et al., Shi et al., Park et al., and Wang et al. Within the trial reported by Cai et al., one single surgeon performed all laparoscopic surgeries and two other surgeons performed the open approach procedures. With regard to the number of cases, surgeons had performed previously, there is no information available. Therefore, there is an unclear risk regarding the learning curve. However, the fact that surgeons are different in both groups also poses a high risk of bias.

### Effects of interventions

The results are partially summarized in a “summary of findings” table (Table 2).

**Table 2:** Summary of findings

Outcomes	Anticipated absolute effects (95% CI)		Relative effect (95% CI)	No. of participants (studies)	Certainty of the evidence (Grade)
	Risk with open gastrectomy	Risk with laparoscopic gastrectomy			
Postoperative serious adverse events	27 per 1000	27 per 1.000 (14 to 55)	RR 1.0100 (0.5027 to 2.0471)	1999 (4 RCTs)	⨁⨁◯◯ Low^a^
Anastomotic leakage	8 per 1000	15 per 1.000 (4 to 59)	RR 1.83 (0.47 to 7.04)	1899 (4 RCTs)	⨁◯◯◯ Very low^a, b, c^
Length of hospital stay	The mean length of hospital stay was 10.14 days	The mean length of hospital stay in the intervention group was 0.81 days fewer (2.58 fewer to 0.96 more)	–	(5 RCTs)	⨁⨁◯◯ Low^a,d^
Incision-to-closure time	The mean incision-to-closure time was 19411 min	The mean incision-to-closure time in the intervention group was 49.1 min higher (17.29 higher to 80.91 higher)	–	(5 RCTs)	⨁⨁⨁◯ Moderate^d^
Intraoperative blood loss	The mean intraoperative blood loss was 145.26 ml	The mean intraoperative blood loss in the intervention group was 42.38 ml fewer (98.73 fewer to 13.98 more)	–	(4 RCTs)	⨁⨁◯◯ Low^a, d^
Harvested lymph nodes	The mean harvested lymph nodes was 34.64	The mean harvested lymph nodes in the intervention group was 0.73 fewer (1.89 fewer to 0.43 more)	–	(5 RCTs)	⨁⨁⨁⨁ High

^a^Wide confidence intervals

^b^Bias due to learning curve issues

^c^Small number of events

^d^Strong evidence for statistical heterogeneity

#### Primary outcomes

The number of postoperative serious adverse events within 30 days following surgery was reported in four of the included trials [22–25]. Park et al. classified the severity of adverse events using the ASCPC classification, whereas the Clavien–Dindo classification was used in the remaining trials. Serious adverse events were defined as Clavien–Dindo grade 3 or higher or severe complications according to the ASCPC classification system.

**Fig. 3:**
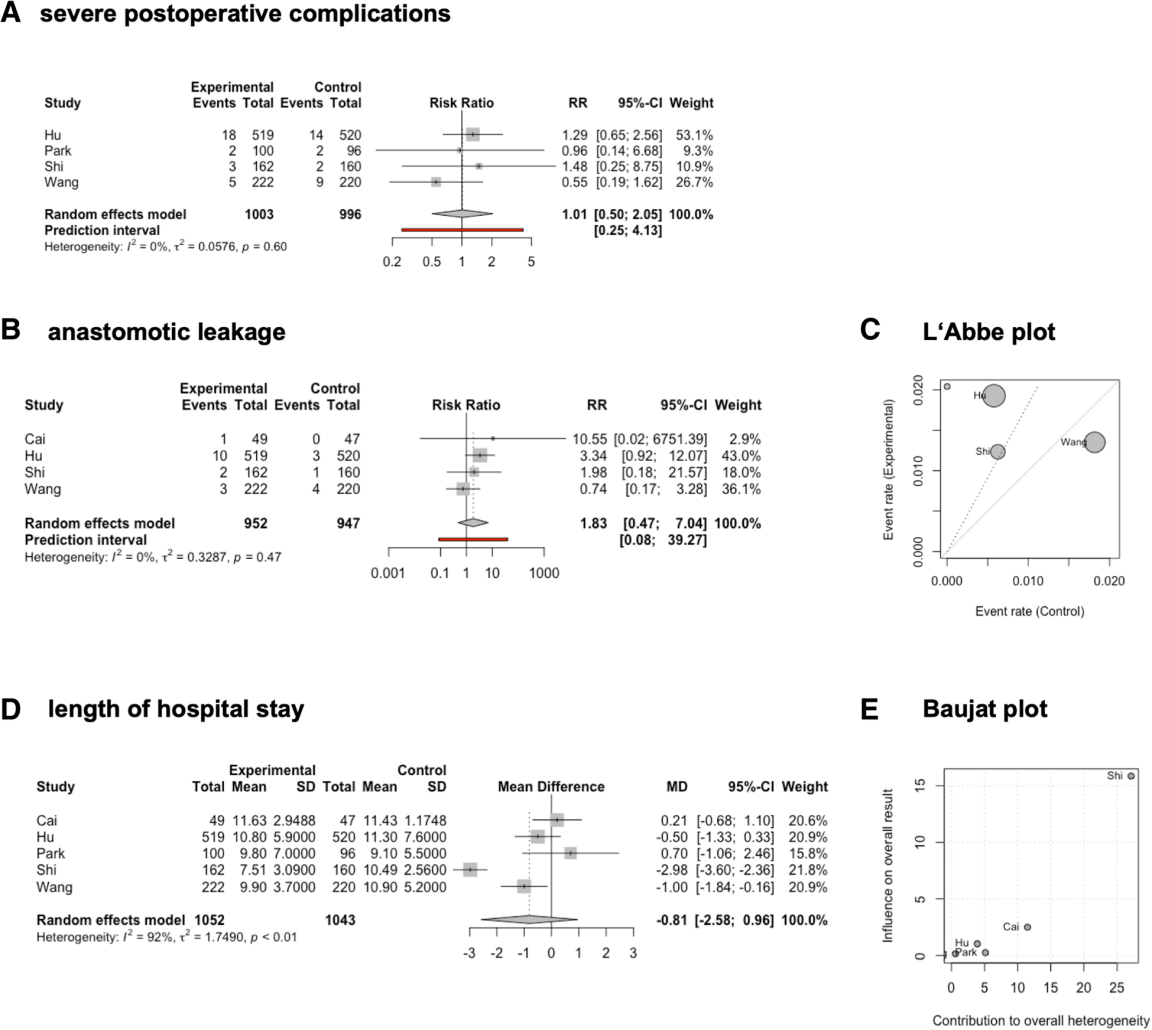
Forest plots of the postoperative serious adverse events (**a**), anastomotic leakage (**b**), and length of hospital stay (**d**) in laparoscopic versus open gastrectomy for locally advanced gastric cancer. **c** L’Abbe plot for the anastomotic leakage. **e** Baujat plot for the length of hospital stay

A meta-analysis of serious adverse events showed that the number of severe postoperative complications occurring within 30 days following surgery was similar in the two groups (RR 1.01, 95% CI 0.5027–2.0471, *p* = 0.9522, tau^2^ = 0.0576, *I*^2^ = 0.0%; Fig. 3a). However, the CIs were wide and overlapped no effect as well as clinically significant effects, indicating a high risk for imprecision of data.

Data on the rate of anastomotic leakage was provided in four of the five included trials [19, 22, 24, 25]. The relative risk for anastomotic leakage was not statistically significantly different between the groups (RR 1.83, 95% CI 0.47–7.04, *p* = 0.25, tau^2^ = 0.33, *I*^2^ = 0.0%; Fig. 3b). However, it must be borne in mind that the requirements placed on operating surgeons vary considerably between studies. While in most studies, possible effects of the learning curve on D2 lymphadenectomy have been minimized by monitoring the quality of the lymphadenectomy, there is no technical standard and no quality control on performing the anastomosis in most studies. Thus, it must be assumed that the results of all included studies may be distorted by influences of the learning curve. Of these, the trial reported by Wang et al. displays the least amount of risk, as the number of gastrectomies that had to have been performed previously was highest in this trial. Consistent with that, a L’Abbé plot clearly shows that in all trials except for the trial reported by Wang et al., the risk for anastomotic leakage tend to be increased when using the minimally invasive approach (Fig. 3c). When considering the trial reported by Wang et al., there was no trend observed regarding an increased occurrence of anastomotic leak in the laparoscopic group (1.4% vs. 1.8%, *p* = 0.72 [25]). Consequently, the confidence intervals are very wide and the number of events is small, resulting in a very low grade of evidence (Table 2).

All included trials provided data on postoperative mortality within 30 days following surgery. In three of the five trials, mortality was 0% for both groups. A meta-analysis of the remaining two trials [22, 23] revealed no differences in short-term mortality between the groups (RR 1.40, *p* = 0.24).

All included trials reported on length of hospital stay. The length of hospital stay tended to be lower in the minimally invasive group but not in a manner that reached statistical significance (MD −0.81 days, 95% CI −2.58 to 0.96, *p* = 0.27, tau^2^ = 1.75, *I*^2^ = 91.7%; Fig. 3d). As the test for heterogeneity indicated statistical heterogeneity (*Q* = 48.25, *p* < 0.0001), sensitivity analyses were performed by removing individual studies from the dataset and analyzing the effect of such on the overall results. As shown in a Baujat plot, the trial reported by Shi et al. had a strong influence on heterogeneity as well as the overall effect (Fig. 3e). Therefore, removing the trial by Shi et al. resulted in a decrease in heterogeneity as well as effect size.

None of the included trials provided data regarding health-related quality of life.

#### Secondary outcomes

**Fig. 4:**
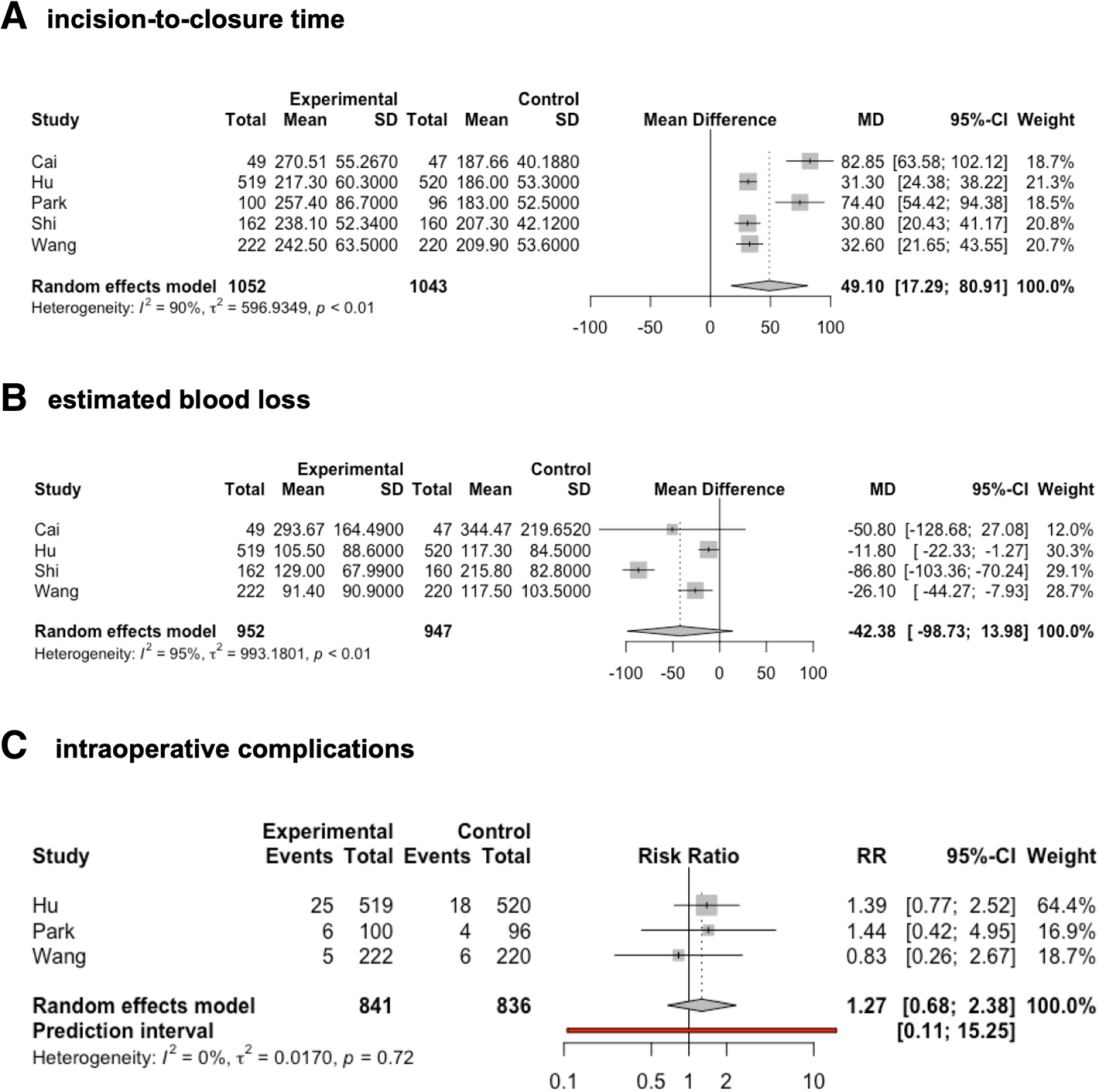
Forest plots of the incision to closure time (**a**), estimated blood loss (**b**), and intraoperative complications (**c**) are shown. Minimally invasive gastrectomy for locally advanced gastric cancer was compared to open gastrectomy

Data on incision-to-closure-time were provided in all of the included trials. In all trials, the minimally invasive approach led to significantly increased operating times. A meta-analysis of these data confirmed this effect resulted in a MD of 49.1 min (95% CI 17.29–80.91, *p* = 0.01, tau^2^ = 596.93, *I*^2^ = 90%; Fig. 4a).

As shown in a Baujat plot, the trials reported by Cai et al. and Park et al. had the strongest influence on both heterogeneity and the overall effect. As a result of the statistical heterogeneity, the increased incision-to-closure time when using the laparoscopic approach is supported by moderate evidence.

Data on estimated intraoperative blood loss were provided for the trials reported by Cai et al., Hu et al., Shi et al., and Wang et al. [19, 22, 24, 25]. Within the studies reported by Hu et al., Shi et al., and Wang et al., estimated blood loss was significantly reduced following the minimally invasive approach, whereas this effect was not statistically significant in the trial reported by Cai et al. Due to the significant heterogeneity of the data (tau^2^ = 993.18, *I*^2^ = 94.7%, *p* < 0.0001), this effect did not reach statistical significance according to the random-effects model (MD −42.38 ml, 95% CI −98.7322 to 13.9769, *p* = 0.097; Fig. 4b). As a result of wide CIs and statistical heterogeneity, this finding is supported by low evidence. A Baujat plot as well as an influence analysis revealed that the trial reported by Shi et al. had the strongest influence on heterogeneity and the smallest influence on the overall effect.

As an additional measure for blood loss, the rate of patients requiring blood transfusions during surgery was analyzed. These data were provided in the trials reported by Cai et al., Hu et al., Shi et al., and Wang et al. [19, 22, 24, 25]. However, whereas Hu et al., Wang et al., and Shi et al. reported the number of patients requiring transfusion, Cai et al. expressed the need for blood transfusion as the mean and standard deviation of the volume of blood transfused intraoperatively. A meta-analysis of pooled data did not show any differences between the two groups (OR 0.826, 95% CI 0.589–1.160, *p* = 0.271).

Regarding the number of patients with intraoperative complications, data were provided in only three trials [22, 23, 25], and a meta-analysis of these data revealed no differences between the two approaches (RR 1.27, 95% CI 0.68–2.38, *p* = 0.244, tau^2^ = 0.02, *I*^2^ = 0.0%; Fig. 4c).

The number of patients needing reoperation was provided by Hu et al., Park et al., Shi et al., and Wang et al. A meta-analysis of these data did not show any difference between the minimally invasive and open approach (RR 0.85, 95% CI 0.32–2.30, *p* = 0.65, tau^2^ = 0.33, *I*^2^ = 0.0%).

**Fig. 5:**
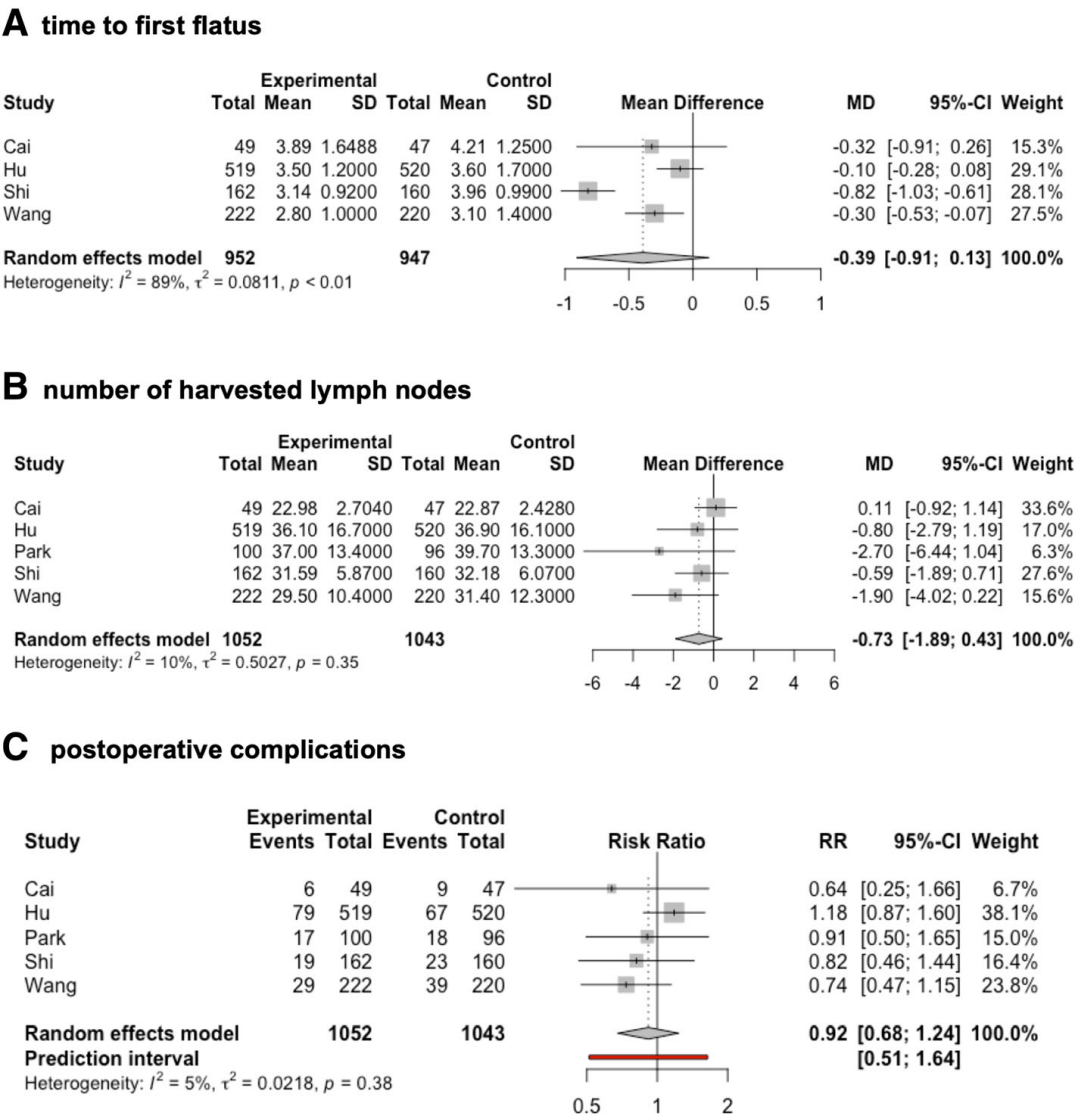
Forest plots time to the first flatus (**a**), number of resected lymph nodes (**b**), and postoperative complications (**c**) are shown. Minimally invasive gastrectomy for locally advanced gastric cancer was compared to open gastrectomy

Time to first flatus was reported in the publications of Cai et al., Hu et al., Shi et al., and Wang et al. In all trials, time to first flatus was decreased in the laparoscopic group. Therefore, the meta-analysis of these data revealed a trend for a decrease of time to first flatus for laparoscopic gastrectomy (MD −0.39 days, 95% CI −0.91 to 0.13; Fig. 5a). However, this effect did not reach statistical significance due to the heterogeneity of the data (tau^2^ = 0.08, *I*^2^ = 88.9%, *p* < 0.0001). A Baujat plot followed by an influence analysis indicated a strong effect of the trial reported by Shi et al. on heterogeneity as well as on the effect size. In addition to statistical heterogeneity, the CIs were wide and overlapped no effect as well as clinically significant effects, indicating a high risk for imprecision of data resulting in a low evidence.

The number of harvested lymph nodes was investigated in all included trials. The minimally invasive approach did not alter the number of resected lymph nodes (MD −0.73, 95% CI −1.89 to 0.43, *p* = 0.16, tau^2^ = 0.50, *I*^2^ = 10.3%; Fig. 5). Remarkably, this was the only result that was not distorted by a significant risk of bias. Thus, the fact that the laparoscopic approach does not compromise lymphadenectomy is the only result supported by high evidence.

Compliance to D2 lymphadenectomy was reported within the trials of Hu et al. and Park et al.; the former detected compliance to D2 lymphadenectomy using video documentation of the operative field following lymphadenectomy and achieved compliance with D2 lymphadenectomy in 99.4% for laparoscopic and 99.6% for open gastrectomy procedures, respectively.

Separately, Park et al. evaluated D2 lymphadenectomy using a checklist for unedited video review. Videos were reviewed by three randomly assigned investigators.

These authors reported compliance with D2 lymphadenectomy in 53% of laparoscopic and 56.8% of open gastrectomy procedures without a significant difference.

All trials included in this meta-analysis provided data on the number of patients suffering from any complication in the 30 days following surgery. In four of the five trials included in this meta-analysis, morbidity tended to be decreased in the laparoscopic group. However, this effect did not reach significance in any of the trials. In contrast, the trial reported by Hu et al. detected morbidity rates of 15.2% in the laparoscopic group versus 12.9% in the open group (*p* = 0.285 [22]). A meta-analysis of these data revealed no difference in the risk of postoperative complications for the minimally invasive approach versus the open approach (RR 0.92, 95% CI 0.68–1.24, *p* = 0.46, tau^2^ = 0.02, *I*^2^ = 5.4%; Fig. 5c).

Pulmonary complications were separately investigated in the trials reported by Cai et al., Hu et al., Shi et al., and Wang et al. [22, 24, 25]. A meta-analysis of these data did not show any statistically significant differences (RR 0.80, 95% CI 0.25–2.52, *p* = 0.57, tau^2^ = 0.32, *I*^2^ = 36.2%).

Wound complications were separately investigated in four of the included trials [19, 22, 24, 25]. None of the included trials showed a significant change in the rate of wound complications for patients who underwent the minimally invasive approach. Consequently, the meta-analysis of respective data showed no differences between the two groups (RR 0.79, 95% CI 0.21–3.05, *p* = 0.62, tau^2^ = 0.29, *I*^^^2 = 6.1%).

None of the studies provided data on the return to normal life activity or return to work.

Overall survival at the time of maximum follow-up was reported in only one trial, with rates of 67.1% for the minimally invasive group and 53.8% for the open group; this difference was not significant [19].

Data for 3-year disease-free survival were provided in the report by Park et al. [31], with no differences found between the study groups (*p* = 0.4477). Reports of the remaining trials only investigated early outcomes, with late results pending. There were no further reports of long-term results within the included trials.

## Discussion

Several RCTs to date have been conducted comparing laparoscopic distal gastrectomy to open distal gastrectomy for gastric cancer [5, 14, 32–34]. Prior meta-analyses of these trials have suggested improved short-term outcomes, particularly for length of hospital stay for the laparoscopic approach, which was balanced by the time-consuming and technically challenging nature of the procedure [35, 36]. However, a recent Cochrane Review stated that, secondary to wide CIs and the substantial heterogeneity of the data, differences in length of hospital stay need to be investigated further. However, there were no statistically significant differences in short-term mortality, long-term mortality, rate of serious adverse events within 3 months of surgery, rate of recurrence within 6 months, rate of recurrence after 6 months, amount of blood transfusions required during or within a week of surgery, rate of any adverse event within 3 months of surgery, quantity of perioperative blood transfused, rate of positive resection margins at histopathological examination, or number of lymph nodes harvested [5].

Nevertheless, most available data are limited to patients suffering from early-stage gastric cancer. Additionally, most trials were conducted in East Asian countries, and the majority were restricted to including patients with distal gastrectomy. A recent meta-analysis of randomized controlled studies including patients suffering from early gastric cancer found that laparoscopic-assisted distal gastrectomy showed beneficial effects on hospital stay and rates of both long-term and short-term complications. However, when D2 lymphadenectomy was performed, the number of harvested lymph nodes was lower when using the laparoscopic approach [37]. While this did not impact oncological outcomes, as relapse rates were comparable between the groups [37], the number of lymph nodes harvested could be more important for locally advanced gastric cancer. For this tumor stage, the current evidence is mostly restricted to case-control and cohort studies. A recent meta-analysis of retrospective trials showed equal numbers of harvested lymph nodes, equal recurrence rates, and comparable cancer-related mortality rates when comparing open distal gastrectomy to the laparoscopic approach [38].

During the last year, three RCTs have been published on the impact of the laparoscopic approach for the treatment of locally advanced gastric cancer [23–25], resulting in a total of five RCTs being available as full-text publications and which provide data specifically for locally advanced gastric cancer.

The present meta-analysis investigated evidence from RCTs of laparoscopic treatment of locally advanced gastric cancer. D2 lymphadenectomy was the standard procedure used in all five trials.

The present meta-analysis based on these five RCTs revealed similar short-term outcomes for laparoscopic versus open resection for locally advanced gastric cancer. However, due to large CIs and substantial heterogeneity between the trials, the quality of evidence for most outcomes remains moderate or low. In contrast, the fact that the laparoscopic procedure does not impair D2 lymphadenectomy is supported by high evidence.

Furthermore, whereas operative time was significantly longer for the laparoscopic group, the minimally invasive approach did not lead to any significant change in the rate of wound complications. Due to data heterogeneity, this meta-analysis could not show any significant effects of the surgical approach on estimated blood loss, need for blood transfusion, or length of hospital stay. These findings are similar to those of a recent Cochrane Review including patients with both early- and advanced-stage gastric cancer [5].

In contrast to a recent meta-analysis including patients with early gastric cancer [37], this meta-analysis clearly shows that the number of harvested lymph nodes did not differ between the laparoscopic and open groups for patients suffering from locally advanced gastric cancer. Thus, the difference of means was less than one lymph node. However, data on long-term oncological outcomes are limited. Thus, overall survival at maximum follow-up was reported in only one trial and was 67.1% for the minimally invasive group and 53.8% for the open group, with no significant differences [19]. Data on 3-year disease-free survival were provided in the report by Park et al. [31], with no differences noted between the study groups (*p* = 0.4477). The existing reports of the remaining trials only investigated early outcomes, with late results still pending. There remains a paucity of evidence regarding long-term outcomes between these approaches. Nevertheless, the apparent extent of resected lymph nodes indicates oncological equivalence of both methods.

As shown in this meta-analysis, the current evidence on laparoscopic gastrectomy has several limitations: first, it is most important to note that laparoscopy-assisted gastrectomy was performed in all included trials and that these data are not fully applicable to totally laparoscopic surgery. Second, most patients included in the present meta-analysis were treated by distal gastrectomy. As the anastomotic technique is particularly challenging in laparoscopic total gastrectomy, the number of anastomosis complications may be underestimated in this meta-analysis. This is supported by a different recent meta-analysis that included high-quality, case-control studies comparing laparoscopic to open total gastrectomy. In this prior meta-analysis, the risk of anastomotic complications was slightly increased in the laparoscopic group, but this trend did not reach a level of significance [39].

Third, all of the included trials were conducted in East Asian countries; thus, these data are not fully transferrable to patients from Western countries. As an analysis of cohorts of Korean and the United States (US) patients suffering from gastric cancer shows, age and body mass index are significantly higher in the US patients, while tumors are more often localized distally in Korean patients [40]. Consequently, total gastrectomy is necessary more often in patients from Western countries. A fourth, very important point for locally advanced cancer is that multimodal treatment strategies for gastric cancer differ between the East and West. Whereas perioperative chemotherapy is the standard in Western countries, locally advanced gastric cancer is typically treated with adjuvant chemotherapy in Eastern countries [41]. Consequently, none of the patients included in this meta-analysis received chemotherapy or radiochemotherapy preoperatively. In a case-control study from a Western population, patients treated with laparoscopy-assisted gastrectomy more often received adjuvant chemotherapy when indicated [42] versus patients treated with an open approach. This finding is of particular importance for patients from the West, as their tumors are on average more advanced and their survival rates from gastric cancer treated by surgery alone are generally worse versus Eastern populations [43]. Adjuvant chemotherapy is standard for locally advanced gastric cancer in Eastern countries; correspondingly, three of the five included studies listed the administration of postoperative chemotherapy within the study protocol. However, most studies did not provide data as to the number of patients who actually received chemotherapy. Additionally, a further limitation is the lack of long-term data from three of the five included studies.

Therefore, in addition to long-term results, further prospective randomized studies from Western countries are necessary.

## Conclusion

Data from five RCTs suggested that overall short-term mortality and morbidity are not impaired by a minimally invasive approach for gastrectomy for locally advanced gastric cancer as compared with the standard open technique. Long-term oncological results cannot be evaluated at present, as adequate data are missing. However, the laparoscopic approach does not impair D2 lymphadenectomy, indicating oncological equivalence to the open approach. Further studies are required to investigate whether there are really no advantages for the use of the minimally invasive approach for the management of advanced gastric cancer.

### Additional file

Additional file 1: PRISMA 2009 Checklist. (DOC 63 kb)

## Appendix 1

**Table 3:** PICOS criteria for inclusion and exclusion of studies

Parameter	Inclusion criteria	Exclusion criteria
Patients	• Adults ≥ 18 years undergoing gastrectomy with D2 lymphadenectomy for locally advanced gastric adenocarcinoma	• Patients under 18 years of age • Patients undergoing gastrectomy for reasons other than gastric adenocarcinoma
Intervention	• Laparoscopic or laparoscopically assisted surgery	• Hand-assisted surgery • Robotic surgery
Comparator	• Open surgery	
Outcomes	• Studies including at least one of the following primary outcome measures: - Short-term mortality - Rate of anastomotic leakages - Postoperative serious adverse events within 30 days after surgery - Length of hospital stay - Health-related quality of life within three and 12 months after surgery	• Studies not including at least one of the primary outcome measures
Study design	• Randomized controlled trials • At least 30 days of follow-up	• Trials that are not randomized controlled trials • Shorter follow-up than 30 days

## Appendix 2

**Table 4:** Search strategy: MEDLINE (PubMed)

Recent queries in PubMed		
Search	Query	Items found
#3	Search (#2) AND ("1980/01/01"[Date - Publication]: "2018/12/31"[Date - Publication])	3064
#2	Search (#1) AND (laparosc* OR "minimally-invasive")	3120
#1	Search ((gastrectomy) AND (gastric OR stomach)) AND (cancer OR carcinoma)	20646

## Appendix 3

**Table:** **Table 5**

Author	Published year	References	Inclusion criteria	Exclusion criteria
Cai	2011	Cai, Wei et al. [19]	Gastric cancer Potentially resectable local tumors without distant metastatic Tumors which need to be controlled by operation against bleeding and obstruction	Patients needed thoraco-abdominal surgery Patients with other malignant tumors History of major upper abdominal surgery Gastric stump cancer Recurrent cancer ASA > III Cardiovascular risk greater than New York Heart Association grade II Severe liver disease (Child B or C) Renal dysfunction.
Hu	2016	Hu, Huang et al. [22] Hu, Huang et al. [26]	Patients aged 18 to 75 years Gastric adenocarcinoma proven by endoscopic biopsy cT2-4aN0-3M0 at preoperative evaluation according to AJCC Cancer Staging Manual, 7th Edition Expected curative resection via distal subtotal gastrectomy with D2 lymphadenectomy ECOG status 0 or 1 ASA I, II, or III Written informed consent	Pregnant or breastfeeding women Severe mental disorder Previous upper abdominal surgery (except laparoscopic cholecystectomy) Previous gastrectomy, endoscopic mucosal resection, or endoscopic submucosal dissection Enlarged or bulky regional lymph node diameter larger than 3 cm based on preoperative imaging Other malignant disease within the past 5 years Previous neoadjuvant chemotherapy or radiotherapy Unstable angina, myocardial infarction, or cerebrovascular accident within the past 6 months Continuous systematic administration of corticosteroids within 1 month before the study Requirement of simultaneous surgery for other diseases Emergency surgery due to a complication (bleeding, obstruction, or perforation) caused by gastric cancer FEV1, 50% of predicted values
Park	2017	Park, Yoon et al. [31] Kim, Park et al. [27] Kim, Park et al. [20] Nam, Kim et al. [21]	Patients aged 20 to 80 years Histologically proven adenocarcinoma of the stomach Clinical stage cT2-T4a cN0-3	Participation in another trial interfering with the outcome of the present study Language problems Lack of compliance Mental inability Synchronous or previous malignant disease (except curatively treated in situ cervical cancer or curatively resected nonmelanoma skin cancer) Systemic administration of corticosteroids, Unstable angina or myocardial infarction within 6 months Severe respiratory disease ASA score > 3 Previous major abdominal surgery Previous chemo- or radiotherapy Inadequate liver, kidney, and bone-marrow functions ECOG status > 1
Shi	2018	Shi, Xu et al. [24]	Patients aged 18–80 years Gastric adenocarcinoma proven by endoscopic biopsy Preoperative cancer stage cT2-3N0-3M0 (according to AJCC-6th TNM staging)	Pregnancy ASA score > 3 Severe mental disorder Surgical history of upper abdomen (except laparoscopic cholecystectomy) Presence of other malignancies History of chemotherapy or radiation therapy Unstable angina or myocardial infarction within the past 6 months FEV1 less than 50% of predicted value Abdominal wall hernia Diaphragmatic hernia Coagulation disorder Portal hypertension
Wang	2018	Wang, Xing et al. [25]	Patients age ≥ 18 years pathologically confirmed primary gastric adenocarcinoma proven by endoscopic biopsy Tumor located in the lower part of the stomach, potentially resectable by subtotal gastrectomy and D2 lymph node dissection Preoperative cancer stage cT2-4aN0-3M0 (according to AJCC-7th TNM staging) ECOG status of 0 or 1, or the American Society of Anesthesiology Classes of I, II, or III Signed informed consent	Surgical history of upper abdomen (except laparoscopic cholecystectomy) Previous gastrectomy, including endoscopic submucosal dissection and endoscopic mucosal resection Integrated or enlarged lymph node with maximum diameter larger than 3 cm according to preoperative imaging Other malignant diseases (within 5 years) Preoperative chemotherapy, immunotherapy, or radiotherapy Other illnesses needed operation concurrently Complications (bleeding, perforation, or obstruction) required emergency surgery due to primary gastric malignancy FEV1 less than 50% of predicted value Patient suffered from bleeding tendency disease such as hemophilia or took anti-coagulant medication due to deep vein thrombosis

## Appendix 4

**Table 6:** Surgeons' qualification and control measures for surgical quality within the trials included in the meta-analysis

Author	year	references	Surgeons‘ qualification	Quality control
Cai	2011	Cai, Wei et al. [19]	One single surgeon for the laparoscopic approach Two other surgeons for the open approach	N/a
Hu	2016	Hu, Huang et al. [22] Hu, Huang et al. [26]	Surgeons Have performed at least 50 distal gastrectomies with D2 lymphadenectomy using open and laparoscopic approaches Were determined to be qualified surgeons by the CLASS academic committee on the basis of the evaluation of unedited videos of both their open and laparoscopic gastrectomy with D2 lymphadenectomy procedures Institution At least 300 gastrectomies for patients with AGC annually at each institute	Surgical quality control was maintained by using mandatory intraoperative photographs that identified specific surgical fields, the resection margin of the specimen, and the abdominal incision Five photos were required to verify the surgical quality of the D2 lymph node clearance as follows: (1) The area between the pancreatic tail and the lower pole of the spleen (2) The pancreatic head and infrapyloric area (3) The right side of the suprapancreatic area (4) The left side of the suprapancreatic area (5) The lesser curvature area These photos were reviewed, and feedback on the assessment was regularly provided to the investigators
Park	2018	Park, Yoon et al. [31] Kim, Park et al. [27] Kim, Park et al. [20] Nam, Kim et al. [21]	Surgeons Had performed at least 30 LADG procedures before the start of this study	To standardize the open and laparoscopic D2 lymphadenectomy procedures, all surgeons attended 10 video seminars to observe unedited videos of the surgical procedure before the start of this trial. To evaluate the D2 lymphadenectomies, we created a list of checkpoints to determine their success.
Shi	2018	Shi, Xu et al. 2017	Surgeons Had performed either LAG or OG with D2 lymphadenectomy in more than 50 cases Before the trial, all participating surgeons reviewed and agreed to the technical details for the surgical procedures Institution Center with significant experience in gastric cancer surgery	Surgical quality control was maintained by regular reviews of the recorded videos of LAGs and the photographs of OGs Results of the assessments were provided to each surgeon
Wang	2018	Wang, Xing et al. 2018	Surgeons Specialized in gastric surgery Have already conducted at least 60 ODG and 60 LADG with D2 lymphadenectomy previously Institutions At least 80 gastrectomies/institution for advanced gastric cancer patients each year	Intraoperative photographs and unedited videos were mandatory required and monitored by the study chair to control the surgical quality Ten photos were uploaded for each participant. Among them, five pictures were taken for lymph node dissection fields, four for the lesion and resection margins of specimens, and one for the abdominal incision
